# The Mediation Effects of Perceived Healthcare Discrimination on Racial and Ethnic Disparities in End of Life Care

**DOI:** 10.1093/geroni/igab046.3448

**Published:** 2021-12-17

**Authors:** Junghee Han

**Affiliations:** University of Southern Indiana, Evansville, Indiana, United States

## Abstract

Numerous studies demonstrate racial and ethnic differences in end-of-life (EOL) care, including place of death and hospice use. Experiencing discrimination in healthcare is often cited in the literature as a potent source explaining the pathways of the racial and ethnic disparities in EOL care. However, none of the studies have tested its mediating effects on racial and ethnic disparities in EOL care. The study examines if the effects of race and ethnicity on place of death and hospice use are mediated by perceived healthcare discrimination. This is a secondary analysis of 2008-2014 data from the nationally representative Health and Retirement Study, including oversampling of Blacks and Hispanics. Samples included 1,446 decedents aged 65 or older who completed an exit interview by proxy. Perceived healthcare discrimination was measured by a self-report of receiving poorer service or treatment than other people from doctors or hospitals. In multivariate analyses, Blacks were more likely to die at hospitals (OR=1.57, p < .05) than Whites. Those who experienced discriminatory healthcare were more likely to die at hospitals than those who never experienced discriminatory healthcare (OR=1.44, p < .05). However, the Karlson-Home-Breen (KHB) method showed no significant mediating effects of perceived healthcare discrimination on racial and ethnic disparities in place of death. Race and ethnicity did not affect hospice use. Although there is no mediating effect of healthcare discrimination on racial and ethnic disparities in EOL care, its direct impact is observed. The research contributes to evidence on the significant role of discrimination in healthcare choices.

